# Use of Adipose Stem Cells Against Hypertrophic Scarring or Keloid

**DOI:** 10.3389/fcell.2021.823694

**Published:** 2022-01-06

**Authors:** Hongbo Chen, Kai Hou, Yiping Wu, Zeming Liu

**Affiliations:** Department of Plastic and Cosmetic Surgery, Tongji Hospital, Tongji Medical College, Huazhong University of Science and Technology, Wuhan, China

**Keywords:** hypertrofic scars, adipose derived stem cell, wound healing, stem cell therapy, mechanism, keloid

## Abstract

Hypertrophic scars or keloid form as part of the wound healing reaction process, and its formation mechanism is complex and diverse, involving multi-stage synergistic action of multiple cells and factors. Adipose stem cells (ASCs) have become an emerging approach for the treatment of many diseases, including hypertrophic scarring or keloid, owing to their various advantages and potential. Herein, we analyzed the molecular mechanism of hypertrophic scar or keloid formation and explored the role and prospects of stem cell therapy, in the treatment of this condition.

## Introduction

Hypertrophic scars are abnormal fibroproliferative wound healing reactions (Zouboulis and Orfanos, 1990; Limandjaja et al., 2020), hypertrophic scars can continue to develop into keloids, keloids are lesions that are higher than the skin surface and beyond the original injury range. The texture of the keloid becomes hard and thickened, presenting nodular or flake masses. Hypertrophic scars and keloid are characterized by excessive scar tissue formation and invasive growth beyond the original wound boundary. Hypertrophic scarring is an unpleasant, maladaptive comorbidity that affects most people around the world and imposes a heavy social and economic burden on affected parties.

Scars usually occur on specific body sites, such as the anterior chest, ear-lobe, mandibular border, and the suprapubic region (Huang and Ogawa, 2021). In addition to obvious cosmetic disfigurement, hypertrophic scars can also produce symptoms such as itching, pain, contracture, and movement restriction, resulting in serious impairment of emotional health and reduced quality of life (Bijlard et al., 2017; Balci et al., 2009). Hypertrophic scars can be acquired genetically or pathologically. Histologically, hypertrophic scars are benign hyperplastic disorder caused by excessive accumulation of extracellular matrix (ECM) and other components (Feng et al., 2017).

The incidence of hypertrophic scarring varies widely and is known to correlate with race and ethnicity. Incidence rates are reportedly as low as <1.0% in Taiwanese Chinese and Caucasians (Seifert and Mrowietz, 2009; Sun et al., 2014), and range from 4.5 to 16.0% in the black and Hispanic general population (Rockwell et al., 1989; Hunasgi et al., 2013). More recent data are available for specific subgroups, reporting that the incidence of hypertrophic scar formation increases significantly in African Americans compared to Caucasians, Asians and other groups, after head and neck surgery, and in women, following cesarean section (C-section) (Tulandi et al., 2011; Sun et al., 2014). Overcoming the complications, and social and economic burden caused by hypertrophic scarring, has become an important medical research topic.

## Mechanism of Scar Formation

Scar formation is part of the abnormal wound healing response after non-severe trauma, such as caesarean section incision, chest hair folliculitis, ear piercing and vaccination. The potential mechanism of scar is complicated, involves genetic susceptibility, mechanobiology, endocrine factors, infection, excessive inflammatory response and so on (Huang and Ogawa, 2021; Yu et al., 2021, Riccio et al., 2019b). Scar formation include multiple cell factors and synergy between multiple stages of repair.

The stages of wound healing mainly comprise inflammation, proliferation, and reshaping (Mari et al., 2015; Leavitt et al., 2016; Morikawa et al., 2019; Rodrigues et al., 2019; Tan et al., 2019; De Francesco et al., 2020). When skin lesions occur, the first reaction entails the formation of a platelet plug, followed by clot formation that immediately stems the bleeding. Then, damaged tissue and activated platelets initiate an inflammatory response by recruiting immune cells such as neutrophils and monocytes to fight local infection, engulf localized debris and damaged connective tissue, and subsequently remove fibrin. After the inflammation subsides, the hyperplasia phase begins, during which new blood vessels and connective tissue appear in the wound area. Re-epithelialization, driven by keratinocyte migration, marks the beginning of proliferation. Thereafter, organizational maturity signifies the reshaping phase, which involves the degeneration of neovascularization and concomitant reconstruction of the extracellular matrix (ECM), resulting in development of organized collagen fibrils that serve as the basis of normal scarring (Wang et al., 2020).

Interference with any of these processes can lead to poor or excessive wound healing, resulting in the formation of hypertrophic scars and non-malignant dermal tumors, which share similar phenotypes, cellular bioenergetics, epigenetic methylation, and other characteristics. Platelet-derived growth factor (PDGF), transforming growth factor-β (TGF-β) and other factors can lead to fibroblast dysfunction and phenotypic changes, ECM disorder and repeated deposition, and an unbalanced angiogenesis cascade, ultimately causing excessive scarring (Tan et al., 2019; Wang et al., 2020; Putri and Prasetyono, 2021).

### Genetic Variation, Epigenetic Modifications and Scar

Single nucleotide polymorphisms have also been implicated in keloid formation, and genetic studies have identified several SNPS and genes that may contribute to understanding the association with keloid development (Halim et al., 2012, Tsai and Ogawa, 2019). For example, a genome-wide association study (GWAS) by Nakashima et al. found that four SNPS (rs873549, rs1511412, rs940187 and rs8032158) in three chromosomal regions were significantly associated with keloid (Nakashima et al., 2010). In other study, Ogawa et al. also reported that rs8032158 may also influence keloid development (Halim et al., 2012).

Recent studies suggest that epigenetic inheritance may also contribute to keloid formation. For example, DNA methylation can change the structure of DNA, thus affecting cell differentiation and cell phenotype (Bataille et al., 2012). Other epigenetic changes include changes in cell phenotypes by popular non-coding RNA. Epigenetic modifications and DNA methylation caused by non-coding RNA (such as microRNAs and LncRNAs) may also play an important role by inducing sustained activation of keloid fibroblasts (Tsai and Ogawa, 2019).

### Mechanical Stress and Scar

The two main anatomical layers of skin, from top to bottom, are the epidermis and dermis. The epidermis consists mainly of epithelial keratinocytes, which are forced together by actin (Harn et al., 2016). These keratinocytes form the skin’s barrier and are layered on top of the dermis, acting as building blocks against external shearing and stretching forces. In contrast, the dermis contains mostly ECM, blood vessels, fibroblasts, and other mesenchymal cells, rather than epithelial cells. Mesenchymal cells, such as fibroblasts, exhibit more cell-matrix interactions (Tracy et al., 2016; Harn et al., 2019; Watt and Fujiwara, 2011).

Mechanical forces regulate skin homeostasis and play a role in the pathogenesis of skin diseases (Fu et al., 2021). As mention, the dermis is rich in ECM—especially collagen, secreted by fibroblasts—that provides the bulk of the skin’s tension (Hsu et al., 2018). Changes in external tension and the internal mechanical properties of cells are associated with collagen, and scarring is specifically the result of excessive collagen production. To some extent, the mechanical stiffness of the skin environment determines the regenerative ability of wound healing. Strategies that alter mechanical forces or mechanical transduction signals may provide new approaches for treating skin diseases and promoting skin regeneration (Harn et al., 2019).

### Immunological Aspects of Scar

Scarring and autoimmune skin disease represent fibrosis events that require a variety of growth factors and result from synergy between immune cells and the medium. These conditions share certain features, such as high infiltration of immune cells and immunoglobulin (Ig), as well as complement deposition in the scar tissue and scar fibroblasts, and medium-high secretion of immune factors, among others (Dong et al., 2013; Jiao et al., 2015; Ogawa, 2017).

Scar can be classified as inflammatory, nodular, and mixed types (Yu et al., 2021). The number and type of immune cells in a patient’s scar tissue or peripheral blood may vary by type and stage. Moreover, the formation of a scar may be related to abnormal wound healing and related immune factors, especially in repeatedly recurring scars. Hence, the classification of scar types and stages provides direction for future research. In the process of scar formation, the initial activation of mast cells (MCs) is the key factor inducing chronic inflammation. Further, MCs release mediators that initiate host defense cascades leading to fibrotic processes. (Hsu et al., 2018; Tsai and Ogawa, 2019).

## Current Mainstream Strategies for Inhibiting Scar Formation

Therapeutic strategies for treating hypertrophic scars include steroid injection, surgical hypertrophic scar removal, radiotherapy, compression, and cryotherapy, pulsed dye and CO_2_ laser, silicone gel, nanoparticles and radiotherapy, among others (Salameh et al., 2021; Ogawa, 2021; Svolacchia et al., 2016; Nicoletti et al., 2013; Riccio et al., 2019a; Jimi et al., 2020; Barrera, 2003; Aoki et al., 2020). However, existing treatments are not guaranteed to reduce recurrence of keloids, due to the high recurrence rate thereof at the hypertrophic scar excision site (Coentro et al., 2019; Kumar and Kamalasanan, 2021).

Scar-free healing is the ultimate goal of scar prevention strategies (Falanga, 2005; Ho Jeong, 2010; Heng, 2011; Leavitt et al., 2016). To develop an optimal combination strategy to this effect, it is necessary to fully reveal the causal relationship between key cells and molecules in scar pathogenesis, thus providing new therapeutic targets for scar-free healing. In addition, advances in stem cell and tissue engineering have brought more alternative therapies closer to reality. Functionalization of biomaterials through various drugs and growth factors, provides a well-controlled approach to scar therapy (Fuller et al., 2016; Rahimnejad et al., 2017; Wang et al., 2022).

## Mesenchymal Stem Cells and Adipogenic Stem Cells

Mesenchymal stem cells (MSCs) are primarily responsible for the regeneration of damaged cells and can be obtained from a variety of sources, including bone marrow, umbilical cord, and adipose tissue. MSCs are abundant and have recently been attracting increasing research attention as potential treatment options in many diseases. The basic characteristics of MSCs are defined by the International Society for Cell & Gene Therapy (ISCT), as follows: 1) plasticity when cultured under standard conditions; 2) expression of a unique set of surface antigens specified by the ISCT; 3) ability to differentiate into osteoblasts, chondrocytes, and adipose cells (Dominici et al., 2006; De Francesco et al., 2017; Bougioukli et al., 2018).

Adipogenic stem cells (ASCs) are bone marrow MSCs extracted from fat cells (53). Not only do they have the general characteristics of bone marrow MSCs, but they are also easy to obtain. ASCs are found in large numbers in the human body, have high proliferation and self-renewal potential, and other advantages, based on which numerous studies have shown their potential in addressing many diseases, including hypertrophic scarring (Gir et al., 2012; Lee et al., 2012; Strong et al., 2015; O’Halloran et al., 2017; Gardin et al., 2018; Gentile and Garcovich, 2019; Ren et al., 2019; Xiong et al., 2020).

## Discussion of Adipogenic Stem Cells and Hypertrophic Scarring

Existing studies have shown that ASCs’ mechanism of action entails secretion of bioactive factors (including growth factors and cytokines, among others), on the one hand, to promote cell proliferation, differentiation, and migration. On the other hand, exosomes derived from ASCs play a role (Hu et al., 2016; Li et al., 2018; Han et al., 2019; Kucharzewski et al., 2019). Moreover, ASCs can play a direct part in disease management, through their multidirectional differentiation ability, by forming complex hybrid systems with novel treatment materials.

### Directed Differentiation

ASCs are pluripotent stem cells with the ability of self-renewal and multidirectional differentiation. They are not only the precursors of fat cells, but also capable of differentiating into osteoblasts, chondrocytes, muscle cells, and neuronal cells. ASCs have also been shown to differentiate into keratinocytes. These results suggest that ASCs may also differentiate directly into epidermal and dermal cells to promote tissue regeneration and prevent scarring in an injured area, during wound healing (Joshi et al., 2020; Xiong et al., 2020; Putri and Prasetyono, 2021).

### Secretion of Biologically Active Factors

ASCs regulate inflammation facilitated by immune cells, through paracrine bioactive factors, inhibiting scar hyperplasia. Long-term inflammation is a main cause of hypertrophic scar formation. ASCs are known to modulate the activity of inflammatory cells, thereby attenuating periods of excessive and prolonged inflammation. This immunomodulatory activity of ASCs is conducted by paracrine secretion of several anti-fibrotic cytokines, including prostaglandin E2 (PGE2), interleukin (IL)-10, hepatocyte growth factor (HGF), and nitric oxide (NO) (Seo and Jung, 2016). In contrast, mast cells are involved in the regulation of vascular homeostasis and angiogenesis and can upregulate fibroblast proliferation, resulting in excessive collagen synthesis and differentiation of fibroblasts into myofibroblasts. ASCs can further reduce hypertrophic scarring by inhibiting the number and activity of mast cells (Putri and Prasetyono, 2021).

ASCs can also activate various anti-fibrotic molecular pathways through paracrine signaling, regulate the activity of primary fibroblast transforming growth factor, and stabilize the function of fibroblast and keratinocyte receptor sites, to achieve relevant anti-fibrosis goals (Borovikova et al., 2018). In addition, injection of ASCs can reverse the abnormal vascularization pattern of scar tissue and reshape the microvascular structure (Garza et al., 2014; Luan et al., 2016).

### Release of Adipogenic Stem Cell-Derived Exosomes (Adipogenic Stem Cell-Exos)

Exosomes are a subset of small, membranous extracellular vesicles (Han et al., 2018; Jing et al., 2018) with a diameter of approximately 30–150 nm, which originate from entosis and are distributed in many bodily fluids—including blood, urine, cerebrospinal fluid, and bile—under physiological and pathological conditions. Exosomes include a variety of active biological substances such as proteins, DNA, mRNAs, and microRNAs, which can carry complex biological information and release it into target cells (Hessvik et al., 2016; Buratta et al., 2020). In addition, exosomes may exist between MSCs and target cells as paracrine mediators (Valadi et al., 2007; Chiba et al., 2012). Many studies have shown that ASC-derived exosomes (ASC-Exos) play an important role in cell migration, proliferation, and collagen synthesis (Xiong et al., 2020; Putri and Prasetyono, 2021).

For example, characteristics of scarring include collagen receptor rearrangement, activation of myofibroblasts expressing α-smooth muscle actin (α-SMA), and the production and secretion of high levels of TGF-β1 in affected tissues (Beanes et al., 2003). Xu et al. indirectly inhibited PLOD1 levels in ASCs through post-transcriptional regulation, which may significantly improve the anti-corrosion potential of ASCs during wound healing, by changing macrophage polarization and regulating scar formation (Xu et al., 2021). In addition, Wang et al. found that ASC-Exos reduced scar formation mainly by regulating the ratios of type III:type I collagen, TGF-β3:TGF-β1, matrix metalloproteinases-3 (MMP-3):tissue inhibitor of metalloproteinases-1 (TIMP-1), and promoting human dermal fibroblast HDF differentiation. This resulted in improved ECM reconstruction and other implementations (Wang et al., 2017).

### Formation of Complex Hybrid Systems

ASCs can play a role in disease management by forming complex hybrid systems with novel materials. For example, Wang et al. proved that ASCs are capable of multi-potential—including lipogenic, osteogenic, and chondrogenic—differentiation, and developed ASC-Exos-delivering collagen/poly (L-lactide-co-caprolactone) (P (LLA-Cl)) nanoyarns, which conform to a material system that mimics the morphological structure of the natural tissue matrix. With sufficient biocompatibility and mechanical properties, it can effectively promote neovascularization, cell proliferation and tissue regeneration, and simultaneously limit scar formation, collagen deposition, and formation of multi-layer epithelium (Wang et al., 2022). Liu et al. showed that hyaluronic acid (HA) can be used as immobilizing agent in a constructed system of ASC-Exos with HA, to retain exosomes in the wound area and effectively play a role in wound repair. The system activated wound-based HDF activity and increased re-epithelialization, which was expected to reduce scar formation (Liu et al., 2019). In addition, Wang et al. developed an FHE hydrogel-carrier system with stimulus-responsive ASC-Exos, which significantly increased the regeneration of skin appendages and reduced scar tissue formation (Wang et al., 2019). Hector et al. also identified three types of ECM-based biomaterials—Integra™ Matrix Wound Dressing, XenoMEM™, and MatriStem™—that serve as human ASC delivery vehicles, which could inhibit scar formation (Capella-Monsonís et al., 2020).

## Conclusion and Future Direction

ASCs have the advantages of being derived from a large source, offering abundant availability and high proliferative ability, having low immunogenicity in clinical applications, and being safer and more effective. ASCs histologically promote the regeneration of healthy tissue, reduce fibroblasts, and reconstruct collagen, similar to that of normal skin. At the molecular level, ASCs reduce hypertrophic scarring through direct differentiation and paracrine mechanisms. Clinically, they can improve the color, elasticity, texture, thickness, and size of hypertrophic scars. ASCs have a positive effect on alleviating hypertrophic scarring, indicating their potential as a possible treatment approach in this condition, with broad therapeutic prospect.

## Abbreviations

ASCs, adipose stem cells; ECM, extracellular matrix; HGF, hepatocyte growth factor; ISCT, International Society for Cell & Gene Therapy; IL-10, interleukin-10; mscMSCs, Mesenchymal stem cells; NO, nitric oxide; PDGF, Platelet-derived growth factor; PGE2, prostaglandin E2; TGF-β, transforming growth factor-β.
